# *Vital Signs*: Changes in Firearm Homicide and Suicide Rates — United States, 2019–2020

**DOI:** 10.15585/mmwr.mm7119e1

**Published:** 2022-05-13

**Authors:** Scott R. Kegler, Thomas R. Simon, Marissa L. Zwald, May S. Chen, James A. Mercy, Christopher M. Jones, Melissa C. Mercado-Crespo, Janet M. Blair, Deborah M. Stone, Phyllis G. Ottley, Jennifer Dills

**Affiliations:** ^1^Division of Injury Prevention, National Center for Injury Prevention and Control, CDC; ^2^Division of Violence Prevention, National Center for Injury Prevention and Control, CDC; ^3^Office of the Director, National Center for Injury Prevention and Control, CDC.

## Abstract

**Introduction:**

The majority of homicides (79%) and suicides (53%) in the United States involved a firearm in 2020. High firearm homicide and suicide rates and corresponding inequities by race and ethnicity and poverty level represent important public health concerns. This study examined changes in firearm homicide and firearm suicide rates coinciding with the emergence of the COVID-19 pandemic in 2020.

**Methods:**

National vital statistics and population data were integrated with urbanization and poverty measures at the county level. Population-based firearm homicide and suicide rates were examined by age, sex, race and ethnicity, geographic area, level of urbanization, and level of poverty.

**Results:**

From 2019 to 2020, the overall firearm homicide rate increased 34.6%, from 4.6 to 6.1 per 100,000 persons. The largest increases occurred among non-Hispanic Black or African American males aged 10–44 years and non-Hispanic American Indian or Alaska Native (AI/AN) males aged 25–44 years. Rates of firearm homicide were lowest and increased least at the lowest poverty level and were higher and showed larger increases at higher poverty levels. The overall firearm suicide rate remained relatively unchanged from 2019 to 2020 (7.9 to 8.1); however, in some populations, including AI/AN males aged 10–44 years, rates did increase.

**Conclusions and Implications for Public Health Practice:**

During the COVID-19 pandemic, the firearm homicide rate in the United States reached its highest level since 1994, with substantial increases among several population subgroups. These increases have widened disparities in rates by race and ethnicity and poverty level. Several increases in firearm suicide rates were also observed. Implementation of comprehensive strategies employing proven approaches that address underlying economic, physical, and social conditions contributing to the risks for violence and suicide is urgently needed to reduce these rates and disparities.

## Introduction

Firearm homicides and suicides represent persistent and significant U.S. public health concerns. In 2020, 79% of all homicides and 53% of all suicides involved firearms (somewhat higher than during the preceding 5 years, when 73%–75% of all homicides and 50%–51% of all suicides involved firearms each year) (*1*). Although all population groups experience firearm homicides and suicides, some are disproportionately affected. Firearm homicide rates are consistently highest among males, adolescents and young adults, and non-Hispanic Black or African American (Black) and non-Hispanic American Indian or Alaska Native (AI/AN) persons; firearm suicide rates are highest among males, older adults, and non-Hispanic White (White) and AI/AN persons (*1*).

Economic conditions in communities contribute to risk for violence, including firearm-related violence, and related racial and ethnic inequities (*2*). For example, multiple indicators (e.g., income inequality, unemployment, and housing and economic instability) are associated with risk for homicide and suicide (*3*–*5*). Youth firearm homicide and suicide rates have been associated with poverty at the county level (*6*), and the percentage of youths living in conditions of household poverty is higher among racial and ethnic minority populations (*6*). The economic and social challenges associated with the COVID-19 pandemic could have exacerbated such risks (*2*,*7*).

This study examined changes in firearm homicide and firearm suicide rates coinciding with the COVID-19 pandemic in 2020, in conjunction with existing and potentially widening inequities by race and ethnicity and poverty level. The findings in this report can help identify disproportionately affected populations and guide the development and implementation of evidence-based strategies for communities experiencing social and structural conditions contributing to violence and disparities in violence.

## Methods

This study integrated four data sources: National Vital Statistics System mortality data*; National Center for Health Statistics (NCHS)/U.S. Census Bureau bridged-race population estimates^†^; NCHS county urbanization designations^§^; and U.S. Census Bureau county poverty data.^¶^ Firearm homicides were identified by *International Classification of Diseases, Tenth Revision* underlying cause-of-death codes X93–X95 and U01.4 and firearm suicides by codes X72–X74. A small number of records (approximately 0.25%) that were missing decedent race and ethnicity or age were excluded from the study data. Suicide statistics further excluded data for persons aged <10 years, as intent for self-harm can be difficult to ascertain in young children. Rates for specific age groups are reported as crude rates; other rates were age-adjusted to the year 2000 U.S. standard population. Rates involving firearm homicide or suicide counts <20 are not presented because of concerns about statistical instability; this criterion ensures that relative SEs usually do not exceed 23% under the assumption that counts are Poisson-distributed. Rate comparisons between years refer to absolute differences unless relative (percentage) changes are indicated. For simplicity in this report, comparisons are nominal and do not involve formal statistical testing; however, such comparisons are restricted to statistically stable rates. The county urbanization data provide a single static designation (representing the year 2013) for each individual county. The six original designations were collapsed into three broader designations (large metropolitan, small/medium metropolitan, and nonmetropolitan). The poverty data provide a yearly measure for each individual county (percentage of all persons living in poverty). Counties were grouped according to four fixed poverty ranges, each covering approximately one quarter of the overall 2019 U.S. population. Data analysis was conducted using SAS software (version 9.4; SAS Institute).

## Results

From 2019 to 2020, the overall age-adjusted firearm homicide rate increased substantially, from 4.6 to 6.1 per 100,000 persons (relative change = 34.6%) (Supplementary Figure, https://stacks.cdc.gov/view/cdc/116519) (Table 1). Rates increased across all age groups, with the highest rates and increases observed among those aged 10–24 (from 7.0 to 9.8 per 100,000) and 25–44 years (8.2 to 11.0). Rates also increased for both sexes, with a greater increase observed among males (7.6 to 10.4). By race and ethnicity, the highest rates and increases occurred among Black (19.0 to 26.6) and AI/AN populations (6.4 to 8.1). Rates increased across all U.S. Census divisions (relative changes ranged from 24.6% [South Atlantic] to 51.0% [Middle Atlantic]) and across all levels of urbanization (28.5% [nonmetropolitan] to 36.9% [large metropolitan]). Rate differentials are amplified when considering age, sex, and race and ethnicity simultaneously (Supplementary Table 1, https://stacks.cdc.gov/view/cdc/116520). The largest increases in firearm homicide rates were among Black males aged 10–24 (54.9 to 77.3) and 25–44 years (66.5 to 90.6) and among AI/AN males aged 25–44 years (18.9 to 28.7). Among females, the highest rates and largest increases were among those who were Black, aged 10–24 (6.4 to 9.1) and 25–44 years (6.9 to 10.2).

**TABLE 1:** Changes in firearm homicide incidence, by selected sociodemographic factors — United States, 2019–2020

Characteristic	No. (rate*)		Rate change	% Rate change
	2019	2020		
**U.S. population overall^†,§^**	**14,392 (4.6)**	**19,350 (6.1)**	**1.6**	**34.6**
**Age group, yrs^¶^**				
<10	125 (0.3)	175 (0.4)	0.1	40.9
10–24	4,474 (7.0)	6,176 (9.8)	2.7	38.5
25–44	7,154 (8.2)	9,685 (11.0)	2.8	34.6
45–64	2,176 (2.6)	2,767 (3.3)	0.7	28.0
≥65	463 (0.9)	547 (1.0)	0.1	14.7
**Sex^†,§^**				
Female	2,284 (1.4)	2,954 (1.9)	0.4	29.4
Male	12,108 (7.6)	16,396 (10.4)	2.7	35.5
Race and ethnicity**^,§^				
A/PI, non-Hispanic	228 (1.1)	227 (1.0)	−0.0^††^	−4.2
AI/AN, non-Hispanic	172 (6.4)	221 (8.1)	1.7	27.0
Black, non-Hispanic	8,499 (19.0)	11,904 (26.6)	7.5	39.5
Hispanic (any race)	2,301 (3.6)	2,946 (4.5)	0.9	25.8
White, non-Hispanic	3,192 (1.7)	4,052 (2.2)	0.5	28.4
**U.S. Census Bureau division^†,§,§§^**				
New England	209 (1.5)	280 (2.0)	0.5	32.2
Middle Atlantic	1,064 (2.7)	1,594 (4.1)	1.4	51.0
East North Central	2,319 (5.2)	3,410 (7.7)	2.5	47.8
West North Central	845 (4.2)	1,149 (5.7)	1.5	36.6
South Atlantic	3,754 (6.0)	4,681 (7.5)	1.5	24.6
East South Central	1,527 (8.5)	2,056 (11.3)	2.9	33.7
West South Central	2,293 (5.7)	3,030 (7.5)	1.8	31.2
Mountain	829 (3.4)	1,057 (4.4)	0.9	27.3
Pacific	1,552 (3.0)	2,093 (4.0)	1.0	35.2
**Urbanization level^†,§^**				
Large metropolitan	8,688 (4.8)	11,880 (6.6)	1.8	36.9
Small/Medium metropolitan	4,066 (4.3)	5,380 (5.7)	1.4	32.1
Nonmetropolitan	1,638 (4.0)	2,090 (5.1)	1.1	28.5

**Abbreviations:** A/PI = Asian or Pacific Islander; AI/AN = American Indian or Alaska Native.

* Firearm homicides per 100,000 persons.

^†^ Excludes decedent records with missing race and ethnicity or age.

^§^ Rates are age-adjusted to the year 2000 U.S. standard population.

^¶^ Excludes decedent records with missing race and ethnicity.

** Excludes decedent records with missing age.

**^††^** A value of “−0.0” denotes a negative change rounded to the nearest tenth.

^§§^ https://www2.census.gov/geo/pdfs/maps-data/maps/reference/us_regdiv.pdf

The overall age-adjusted firearm suicide rate among persons aged ≥10 years remained nearly level between 2019 and 2020 (7.9 and 8.1 per 100,000 persons, respectively [relative change = 1.5%]) (Supplementary Figure, https://stacks.cdc.gov/view/cdc/116519) (Table 2). More notable age-specific rate increases among persons aged 10–24 (from 4.7 to 5.4) and 25–44 years (7.6 to 8.1) were partially offset by a decrease among those aged 45–64 years (9.4 to 8.8). Firearm suicide rates by sex remained nearly level. By race and ethnicity, the firearm suicide rate among AI/AN persons showed the largest increase (7.7 to 10.9). Considering age, sex, and race and ethnicity simultaneously, rates of firearm suicide increased most notably among AI/AN males aged 10–24 (14.0 to 23.4) and 25–44 years (16.0 to 23.8) (Supplementary Table 2, https://stacks.cdc.gov/view/cdc/116521).

**TABLE 2:** Changes in firearm suicide incidence, by selected sociodemographic factors — United States, 2019–2020

Characteristic	No.* (rate*^,†^)		Rate change	% Rate change
	2019	2020		
**U.S. population overall^§,¶^**	**23,888 (7.9)**	**24,245 (8.1)**	**0.1**	**1.5**
Age group, yrs**				
10–24	2,969 (4.7)	3,393 (5.4)	0.7	14.7
25–44	6,683 (7.6)	7,105 (8.1)	0.4	5.7
45–64	7,863 (9.4)	7,284 (8.8)	−0.6	−6.8
≥65	6,373 (11.8)	6,463 (11.6)	−0.2	−1.5
**Sex^§,¶^**				
Female	3,214 (2.2)	3,108 (2.1)	−0.1	−3.2
Male	20,674 (14.2)	21,137 (14.5)	0.3	2.0
**Race and ethnicity^††,¶^**				
A/PI, non-Hispanic	381 (2.0)	374 (2.0)	−0.0^§§^	−1.9
AI/AN, non-Hispanic	183 (7.7)	267 (10.9)	3.2	41.8
Black, non-Hispanic	1,588 (4.2)	1,852 (4.9)	0.6	14.3
Hispanic (any race)	1,534 (3.0)	1,790 (3.4)	0.4	13.8
White, non-Hispanic	20,202 (10.4)	19,962 (10.4)	−0.0^§§^	−0.3
**U.S. Census Bureau division^§,¶,¶¶^**				
New England	625 (4.4)	587 (4.2)	−0.3	−6.1
Middle Atlantic	1,587 (4.0)	1,561 (4.0)	−0.0^§§^	−1.0
East North Central	3,257 (7.6)	3,252 (7.6)	0.0^§§^	0.5
West North Central	1,882 (9.9)	1,932 (10.2)	0.3	3.2
South Atlantic	5,254 (8.5)	5,359 (8.7)	0.2	2.1
East South Central	2,041 (11.7)	2,103 (12.1)	0.5	3.9
West South Central	3,487 (9.8)	3,593 (10.1)	0.2	2.5
Mountain	2,911 (13.1)	3,078 (13.6)	0.5	3.8
Pacific	2,844 (5.8)	2,780 (5.6)	−0.2	−3.3
**Urbanization level^§,¶^**				
Large metropolitan	10,085 (6.0)	10,136 (6.1)	0.0^§§^	0.3
Small/Medium metropolitan	8,546 (9.5)	8,727 (9.7)	0.2	2.4
Nonmetropolitan	5,257 (12.4)	5,382 (12.8)	0.3	2.6

**Abbreviations:** A/PI = Asian or Pacific Islander; AI/AN = American Indian or Alaska Native.

* Numbers and rates overall and by sex, race and ethnicity, U.S. Census Bureau division, and urbanization level exclude persons aged <10 years.

^†^ Firearm suicides per 100,000 persons.

^§^ Excludes decedent records with missing race and ethnicity or age.

^¶^ Rates are age-adjusted to the year 2000 U.S. standard population.

** Excludes decedent records with missing race and ethnicity.

^††^ Excludes decedent records with missing age.

^§§^ A value of “−0.0” denotes a negative change rounded to the nearest tenth; a value of “0.0” denotes a positive change rounded to the nearest tenth.

^¶¶^ https://www2.census.gov/geo/pdfs/maps-data/maps/reference/us_regdiv.pdf

County-wide poverty conditions varied by race and ethnicity (Table 3). As of 2020, approximately 24% of the U.S. population overall resided in counties classified as the most impoverished, however, approximately 29% of the Hispanic population, 39% of the Black population, and 44% of the AI/AN population resided in these counties. Firearm homicide rates were lowest and increased least at the lowest poverty level (from 2.0 to 2.4 per 100,000 persons) and were higher and showed larger increases at higher poverty levels (e.g., from 7.7 to 10.8 at the highest level). By race and ethnicity, rates were highest and increased most among Black persons at the two highest poverty levels. Associations between poverty and firearm suicide are also evident (Table 4). Yearly rates were lowest at the lowest poverty level and highest at the highest poverty level for the U.S. population overall and among Hispanic, Black, and White persons. The largest rate increases occurred among AI/AN persons at the two highest poverty levels.

**TABLE 3:** Changes in firearm homicide incidence, by race and ethnicity and surrounding poverty level — United States,* 2019–2020

Race and ethnicity group/Poverty range (%)^†^	2019		2020		Rate change	% Rate change
	% of population in poverty range^§^	No. (rate^¶^)	% of population in poverty range^§^	No. (rate^¶^)		
U.S. population overall**						
**<9.1**	**24.7**	**1,494 (2.0)**	**26.7**	**1,972 (2.4)**	**0.4**	**21.9**
**9.1–12.1**	**24.7**	**2,428 (3.2)**	**28.0**	**4,002 (4.6)**	**1.4**	**45.6**
**12.2–14.6**	**25.7**	**4,340 (5.3)**	**21.3**	**5,058 (7.5)**	**2.2**	**41.0**
**>14.6**	**24.9**	**6,130 (7.7)**	**24.0**	**8,318 (10.8)**	**3.1**	**40.0**
**Total**	**100**	**14,392 (4.6)**	**100**	**19,350 (6.1)**	**1.6**	**34.6**
**A/PI, non-Hispanic^††^**						
<9.1	37.6	49 (0.6)	41.4	64 (0.7)	0.1	14.8
9.1–12.1	23.7	53 (1.0)	23.1	50 (1.0)	−0.1	−5.0
12.2–14.6	26.0	71 (1.3)	21.8	68 (1.4)	0.1	5.6
>14.6	12.6	55 (2.0)	13.6	45 (1.4)	−0.6	−28.1
**Total**	**100**	**228 (1.1)**	**100**	**227 (1.0)**	**−0.0^§§^**	**−4.2**
**AI/AN, non-Hispanic^††^**						
<9.1	12.9	8 (—^¶¶^)	16.0	21 (4.7)	—^¶¶^	—^¶¶^
9.1–12.1	20.6	43 (7.6)	23.5	68 (10.8)	3.2	41.6
12.2–14.6	20.9	34 (5.8)	16.8	27 (5.8)	0.0^§§^	0.6
>14.6	45.6	87 (7.3)	43.7	105 (8.8)	1.6	21.5
**Total**	**100**	**172 (6.4)**	**100**	**221 (8.1)**	**1.7**	**27.0**
**Black, non-Hispanic^††^**						
<9.1	16.9	728 (9.6)	15.9	907 (12.7)	3.1	32.0
9.1–12.1	17.8	1,168 (14.5)	23.1	2,163 (20.6)	6.1	41.7
12.2–14.6	27.3	2,559 (20.9)	22.4	3,098 (30.8)	9.8	47.0
>14.6	38.0	4,044 (24.2)	38.6	5,736 (33.6)	9.5	39.1
**Total**	**100**	**8,499 (19.0)**	**100**	**11,904 (26.6)**	**7.5**	**39.5**
**Hispanic (any race)^††^**						
<9.1	17.5	229 (2.0)	19.0	333 (2.6)	0.6	29.0
9.1–12.1	21.6	385 (2.7)	26.1	650 (3.8)	1.1	38.8
12.2–14.6	32.4	850 (4.1)	25.6	921 (5.5)	1.4	34.3
>14.6	28.5	837 (4.7)	29.3	1,042 (5.6)	0.9	19.6
**Total**	**100**	**2,301 (3.6)**	**100**	**2,946 (4.5)**	**0.9**	**25.8**
**White, non-Hispanic^††^**						
<9.1	27.3	480 (0.9)	30.0	647 (1.2)	0.2	22.9
9.1–12.1	27.3	779 (1.5)	30.2	1,071 (1.9)	0.4	24.8
12.2–14.6	23.4	826 (1.8)	19.8	944 (2.6)	0.7	38.5
>14.6	22.0	1,107 (2.7)	20.1	1,390 (3.7)	1.0	39.0
**Total**	**100**	**3,192 (1.7)**	**100**	**4,052 (2.2)**	**0.5**	**28.4**

**Abbreviations:** A/PI = Asian or Pacific Islander; AI/AN = American Indian or Alaska Native.

* Excludes Kalawao County, Hawaii because of missing poverty data.

^†^ For comparability, the county poverty ranges are constant across race and ethnicity groups and years.

^§^ Percentage of indicated group residing in counties within the specified poverty range.

^¶^ Firearm homicides per 100,000 persons; age-adjusted to the year 2000 U.S. standard population.

** Excludes decedent records with missing race and ethnicity or age.

^††^ Excludes decedent records with missing age.

^§§^ A value of “−0.0” denotes a negative change rounded to the nearest tenth; a value of “0.0” denotes a positive change rounded to the nearest tenth.

^¶¶^ Rate or rate change considered statistically unstable because of homicide count <20.

**TABLE 4:** Changes in firearm suicide incidence, by race and ethnicity and surrounding poverty level — United States,* 2019–2020

Race and ethnicity group/Poverty range (%)^†^	2019		2020		Rate change	% Rate change
	% of population in poverty range^§^	No.^¶^ (rate^¶,^**)	% of population in poverty range^§^	No.^¶^ (rate^¶,^**)		
**U.S. population overall** ^††^						
**<9.1**	**24.7**	**4,782 (6.5)**	**26.7**	**5,439 (6.8)**	**0.3**	**4.7**
**9.1–12.1**	**24.7**	**6,353 (8.5)**	**28.0**	**7,191 (8.4)**	**−0.0^§§^**	**−0.0^§§^**
**12.2–14.6**	**25.7**	**5,903 (7.6)**	**21.3**	**5,248 (8.2)**	**0.6**	**7.5**
**>14.6**	**24.9**	**6,850 (9.3)**	**24.0**	**6,367 (8.9)**	**−0.3**	**−3.6**
**Total**	**100**	**23,888 (7.9)**	**100**	**24,245 (8.1)**	**0.1**	**1.5**
**A/PI, non-Hispanic^¶¶^**						
<9.1	37.6	107 (1.5)	41.4	136 (1.8)	0.3	16.7
9.1–12.1	23.7	98 (2.2)	23.1	91 (2.1)	−0.1	−6.5
12.2–14.6	26.0	111 (2.2)	21.8	95 (2.3)	0.0^§§^	1.3
>14.6	12.6	65 (2.7)	13.6	52 (2.0)	−0.7	−27.4
**Total**	**100**	**381 (2.0)**	**100**	**374 (2.0)**	**−0.0^§§^**	**−1.9**
**AI/AN, non-Hispanic^¶¶^**						
<9.1	12.9	20 (6.9)	16.0	35 (9.2)	2.3	33.0
9.1–12.1	20.6	48 (9.6)	23.5	64 (10.8)	1.2	12.9
12.2–14.6	20.9	25 (4.9)	16.8	47 (11.3)	6.4	128.8
>14.6	45.6	90 (8.4)	43.7	121 (11.5)	3.1	36.6
**Total**	**100**	**183 (7.7)**	**100**	**267 (10.9)**	**3.2**	**41.8**
**Black, non-Hispanic^¶¶^**						
<9.1	16.9	219 (3.4)	15.9	224 (3.7)	0.2	6.6
9.1–12.1	17.8	285 (4.3)	23.1	427 (4.8)	0.5	12.0
12.2–14.6	27.3	425 (4.1)	22.4	427 (5.0)	0.9	22.1
>14.6	38.0	659 (4.7)	38.6	774 (5.3)	0.6	12.8
**Total**	**100**	**1,588 (4.2)**	**100**	**1,852 (4.9)**	**0.6**	**14.3**
**Hispanic (any race)^¶¶^**						
<9.1	17.5	221 (2.4)	19.0	280 (2.8)	0.4	17.9
9.1–12.1	21.6	304 (2.8)	26.1	483 (3.5)	0.7	26.0
12.2–14.6	32.4	483 (2.9)	25.6	444 (3.3)	0.4	14.2
>14.6	28.5	526 (3.7)	29.3	583 (3.8)	0.2	4.3
**Total**	**100**	**1,534 (3.0)**	**100**	**1,790 (3.4)**	**0.4**	**13.8**
**White, non-Hispanic^¶¶^**						
<9.1	27.3	4,215 (8.2)	30.0	4,764 (8.4)	0.2	3.0
9.1–12.1	27.3	5,618 (10.6)	30.2	6,126 (10.5)	−0.1	−0.9
12.2–14.6	23.4	4,859 (10.6)	19.8	4,235 (11.1)	0.5	4.8
>14.6	22.0	5,510 (13.0)	20.1	4,837 (12.6)	−0.3	−2.6
**Total**	**100**	**20,202 (10.4)**	**100**	**19,962 (10.4)**	**−0.0^§§^**	**−0.3**

**Abbreviations:** A/PI = Asian or Pacific Islander; AI/AN = American Indian or Alaska Native.

* Excludes Kalawao County, Hawaii because of missing poverty data.

^†^ For comparability, the county poverty ranges are constant across race and ethnicity groups and years.

^§^ Percentage of indicated group residing in counties within the specified poverty range.

^¶^ Numbers and rates exclude persons aged <10 years.

** Firearm suicides per 100,000 persons; age-adjusted to the year 2000 U.S. standard population.

^††^ Excludes decedent records with missing race and ethnicity or age.

^§§^ A value of “−0.0” denotes a negative change rounded to the nearest tenth; a value of “0.0” denotes a positive change rounded to the nearest tenth.

^¶¶^ Excludes decedent records with missing age.

## Discussion

The firearm homicide rate in 2020 was the highest recorded since 1994 (*1*). However, the increase in firearm homicides was not equally distributed. Young persons, males, and Black persons consistently have the highest firearm homicide rates, and these groups experienced the largest increases in 2020. These increases represent the widening of long-standing disparities in firearm homicide rates. For example, the firearm homicide rate among Black males aged 10–24 years was 20.6 times as high as the rate among White males of the same age in 2019, and this ratio increased to 21.6 in 2020. Although the overall firearm suicide rate remained relatively unchanged between 2019 and 2020, young persons and some racial/ethnic minority groups experienced increases in firearm suicide. Notably, the largest increase occurred among AI/AN persons, resulting in this group having the highest firearm suicide rate as of 2020. Racial and ethnic minority groups are more likely to live in communities with high surrounding poverty, and firearm homicide and suicide were also associated with poverty. Counties with the smallest proportion of the population living below the poverty line experienced a 22% increase in firearm homicides, whereas all other counties experienced an increase of ≥40%. In 2020, counties with the highest poverty level had firearm homicide and firearm suicide rates that were 4.5 and 1.3 times as high, respectively, as counties with the lowest poverty level.

The findings of this study do not support causal inferences, and reasons for increasing rates and widening inequities are unclear and potentially complex. Several explanations have been proposed, including increased stressors (e.g., economic, social, and psychological) and disruptions in health, social, and emergency services during the COVID-19 pandemic; strains in law enforcement-community relations reflected in protests over law enforcement use of lethal force; increases in firearm purchases; and intimate partner violence (*7*–*10*). The COVID-19 pandemic might have exacerbated existing social and economic stressors that increase risk for homicide and suicide, particularly among certain racial and ethnic communities (*2*). Longstanding systemic inequities and structural racism (*11*) have resulted in limited economic, housing, and educational opportunities associated with inequities in risk for violence and other health conditions among various racial and ethnic groups.

The findings of this study underscore the importance of comprehensive strategies that can stop violence now and in the future by addressing factors that contribute to homicide and suicide, including the underlying economic, physical, and social inequities that drive racial and ethnic disparities in multiple health outcomes. For example, policies that enhance economic and household stability (e.g., temporary assistance to families, child care subsidies, tax credits, housing assistance, and livable wages) can reduce family poverty and other risk factors for homicide and suicide (e.g., family stress and substance use) (*3*,*4*,*12*–*14*). Communities can also implement locally driven approaches that address physical and social environments that contribute to violence and other inequities, with the potential for immediate benefits. Approaches such as enhancing and maintaining green spaces and the remediation of vacant buildings can reduce opportunities for violence and promote positive social interactions. These approaches have been associated with significant reductions in risk for firearm violence (*13*,*15*). For example, a study in a major U.S. city found that restoration of vacant lots (e.g., cleaning up debris or adding vegetation) was associated with significant reductions in firearm assaults, with the largest reduction (29%) in areas with the highest poverty (*15*).

In addition to addressing known drivers of inequities and disparities, it is important for prevention strategies to focus on populations experiencing the highest risks for and rates of violence (*4*,*13*). For example, the comprehensive White Mountain Apache Suicide Surveillance and Prevention System was associated with reduced Apache suicides and attempts (*16*). Community and street outreach programs, like Cure Violence, have shown promising results for multiple outcomes, including firearm violence, by connecting populations at highest risk for violence with community services while reducing tensions and retaliatory actions (*4*,*13*). Hospital-based programs that intervene with victims of violence can have lasting effects on risk for revictimization and perpetration (*17*), and those that intervene with patients at risk for suicide can prevent reattempts (*3*,*18*). Other individual and family therapeutic approaches can lessen harm from exposure to violence and prevent continuation of violence (e.g., Trauma Focused Cognitive Behavior Therapy and Multisystemic Therapy) (*4*). Moreover, many violence prevention programs, such as those that teach coping and problem-solving skills, enhance norms against intimate partner and other violence, prevent substance use and suicide attempts, encourage help-seeking, or provide mentoring and employment opportunities can be implemented more broadly, irrespective of risk (*3*,*4*,*13*).

Approaches that focus on enhancing firearm safety and storage, particularly to protect persons at risk from harming themselves or others, are part of a comprehensive prevention strategy. For example, research suggests that physician counseling paired with provision of a safety device is associated with safer firearm storage practices in the home (*19*). A recent review also concluded that child access prevention laws have been associated with lower rates of youth firearm self-injury, including suicide, and laws preventing firearm ownership by those under domestic violence restraining orders are associated with reductions in intimate partner homicides (*20*). It is important to examine the circumstances and mechanisms (e.g., implementation processes and changes in knowledge or norms) that facilitate the most effective firearm safety approaches (*20*). There is substantial need for additional research to expand the evidence base for programs, policies, and practices that effectively reduce firearm injuries and deaths, and that address inequities in risk for violence and suicide.

The findings in this report are subject to at least four limitations. First, the urbanization and poverty measures are county-wide indicators and thus not specific to any demographic subpopulations. Second, statistically stable rate estimates for certain demographic cross-classifications could not be reported because of small counts. Third, rate estimates by race and ethnicity could reflect underreporting of deaths in the vital statistics data, particularly for AI/AN persons. Finally, the study could not determine why observed increases occurred or whether they are attributable to the COVID-19 pandemic or other causes. Preliminary data for 2021 indicate that firearm homicide incidence during the first half of 2021 was higher than that during the same period in 2020, suggesting that the elevated rate might have persisted; however, further analysis is required (*1*).

The increases in firearm homicide rates and persistently high firearm suicide rates in 2020, with increases among populations that were already at high risk, have widened disparities and heightened the urgency of actions that can have immediate and lasting benefits. State and local governments, community partners, and health care and other service providers can use the best available evidence to implement comprehensive approaches to prevent homicide and suicide, including addressing physical, social, and structural conditions that contribute to violence and disparities.

**Summary**

**What is already known about this topic?**

Firearm homicides and suicides represent important public health concerns in the United States, with substantial inequities by race and ethnicity and poverty level.

**What is added by this report?**

In 2020, coincident with the COVID-19 pandemic, the firearm homicide rate increased nearly 35%, reaching its highest level since 1994, with disparities by race and ethnicity and poverty level widening. The firearm suicide rate, although higher than that for firearm homicide, remained nearly level overall but increased among some populations.

**What are the implications for public health practice?**

Communities can implement comprehensive violence prevention strategies to address physical, social, and structural conditions that contribute to violence and disparities.

## Footnotes

*
https://www.cdc.gov/nchs/products/index.htm

†
https://www.cdc.gov/nchs/nvss/bridged_race.htm

§
https://www.cdc.gov/nchs/data/series/sr_02/sr02_166.pdf

¶
https://www.census.gov/content/dam/Census/library/publications/2020/demo/p30-08.pdf
